# Supplementary material for: Determinants of care-seeking behavior for sexually transmitted infections among sexually active men in East Africa: A multilevel mixed effect analysis
Source: PLoS One. 2024 Sep 5;19(9):e0307755. doi: 10.1371/journal.pone.0307755 (PMC11376515; doi:10.1371/journal.pone.0307755)
Supplement: S1 Table — (DOCX) [file pone.0307755.s001.docx]

Supplementary table 1. A bi-variable analysis of factors associated with STI-related health-seeking behavior among sexually active men in East Africa

| **Variable** | **COR(95%CI)** |
| --- | --- |
|  |  |
| **Individual level characteristics** |  |
| **Age** |  |
| 15-24 | 1 |
| 25-34 | 1.56 (1.28, 1.89) |
| 35-44 | 1.27 (1.02, 1.58) |
| >44 | 1.16 (0.89, 1.51) |
| **Marital status** |  |
| Never married | 1 |
| Ever married | 1.13 (0.96, 1.34) |
| **Educational status** |  |
| No formal education | 1 |
| Primary | 1.21 (0.93, 1.58) |
| Secondary | 1.63 (1.25, 2.13) |
| Higher | 1.08 (0.72, 1.62) |
| **Wealth index** |  |
| Poor | 1 |
| Middle | 0.90 (0.73, 1.12) |
| Rich | 0.85 (0.71, 1.02) |
| **Employment status** |  |
| Not employed | 1 |
| Employed | 1.17 (0.93, 1.47) |
| **Age at first sex** |  |
| ≤ 19 | 1 |
| ≥ 20 | 0.67 (0.56, 0.80) |
| **Number of sex partners excluding spouse, in the last 12 months** |  |
| 0 | 1 |
| 1 | 1.98 (1.65, 2.38) |
| ≥2 | 2.66 (2.12, 3.33) |
| **Ever heard about STI** |  |
| No | 1 |
| Yes | 1.12 (0.44, 2.84) |
| **Ever heard of AIDS** |  |
| **No** | 1 |
| **Yes** | 0.70 (0.43, 1.14) |
| **Ever been tested for HIV** |  |
| No | 1 |
| Yes | 1.16 (0.99, 1.36) |
| **Media exposure** |  |
| No | 1 |
| Yes | 1.24 (1.03, 1.51) |
| **Comprehensive knowledge about HIV/AIDS (n=** **2,838)** |  |
| No | 1 |
| Yes | 1.73 (1.24, 2.43) |
| **Covered by health insurance** |  |
| No | 1 |
| Yes | 0.99 (0.79, 1.24) |
| **Community level characteristics** |  |
| **Residence** |  |
| Urban | 1 |
| Rural | 0.89 (0.75, 1.06) |
