# Supplementary material for: Determinants of care-seeking behavior for sexually transmitted infections among sexually active men in East Africa: A multilevel mixed effect analysis
Source: PLoS One. 2024 Sep 5;19(9):e0307755. doi: 10.1371/journal.pone.0307755 (PMC11376515; doi:10.1371/journal.pone.0307755)
Supplement: Attachment — Submitted filename: A point by point response to reviewers.docx [file pone.0307755.s002.docx]

**Reviewer 1**

Reviewer #1: This manuscript describes a secondary analysis of demographic and health survey data, and assesses the prevalence of STI-related care seeking behaviour, and associated factors. It is an important and interesting topic as treatment for prevalent STIs is an important factor to prevent onward STI transmission.
Overall, the manuscript read relatively well. However, there are several aspects that need to be improved before being suitable for publication. Most importantly, I do not feel that the methods provide enough information to replicate this study, with variable choices and model development being described quite superficially. Additionally, the paper would benefit from several definitions, including for “men who had an STI”, “sex”, “media exposure”, and “STI-related care seeking”.

**Author’s response:** Dear reviewer, Thank you for your patience in reading and practical suggestions! We have addressed all your concerns in the revised manuscript.

Generally, the narrative is clear – however there were multiple mentions of asymptomatic infections, which I do not feel are particularly relevant for STI treatment seeking behaviour. There also needs to be more context within broader literature. In particular, no comparison is made in either introduction or discussion with a paper by Ogale et al. (10.1371/journal.pgph.0001626) that is arguably the most relevant recent evidence assessing STI treatment seeking in adults in Uganda.

**Author’s response:** Thank you, dear reviewer, for your time and constructive comments! We have comprehensively addressed all your comments and suggestions in the revised manuscript.

Finally, there are several broad and/or definitive statements that would benefit from referencing.
Of note, I have made comments about the methodology but would suggest a dedicated statistical assessment, particularly around model development and comparison.

**Author’s response:** Dear reviewer Thank you very much for your valuable, detailed comments!

Abstract
Page 2, lines 28-29: “Despite many STIs being manageable, their often asymptomatic nature poses a significant threat, leading to substantial morbidity and mortality.”
- The mention of the asymptomatic nature of STIs is not that relevant here, given that it assesses care-seeking behaviour in men with symptoms.

**Authors’ response:** Thank you for your professional insight. We have made corrections based on your comment. **(See the revised manuscript, introduction section of the abstract)**

Page 2, line 40: “The pooled prevalence of STI-related care-seeking behavior among sexually active men in East Africa was 71% (95%CI: 69.76, 72.75).”

- A definition of “STI-related care-seeking behavior” is required in the methods section of the abstract.

Author’s response: Dear reviewer thank you for your suggestion. We have included the definition of “STI-related care-seeking behavior” in the study variables section of the method part.
- Please provide odds ratios and confidence intervals for the associations described in the results section of the abstract

**Authors’ response:** Thanks for your comment! We have added it. **(See the revised manuscript, results section of the abstract)**

- The conclusions described in the abstract are not strongly supported by the results. For example, given that ever being tested for HIV is associated with treatment-seeking behaviour, it is unclear why enhancing health promotion and education programmes “especially during HIV testing and counselling” would be particular important? The other recommendations are quite vague and not necessarily linked to the results. For example, although a “holistic approach that involves healthcare providers, policymakers, community leaders, and young men” seems generally sensible, it is not clear how this build on the results presented.

**Authors’ response:** Thank you for your professional insight. We have made corrections based on your comment. **(See the revised manuscript, conclusion section of the abstract)**

Introduction

- Neither the introduction nor discussion references the most relevant piece of recent research on this topic by Ogale et al. (10.1371/journal.pgph.0001626) which assessed STI care seeking in adults in Uganda. I suggest this should be a key focus of comparison in the discussion.

**Author’s response**: Thank you for your professional insight! We have incorporated the suggested study in the revised manuscript. **(See the revised manuscript, Introduction section)**

Page 3, lines 53-54: “Globally, the prevalence of sexually transmitted infections (STIs) is rising despite the adoption of effective preventive measures”
- The phrase "despite the adoption of effective preventive measures" is both broad and vague. It is unclear what preventive measures are being referred to. At minimum, a reference is required to support this statement.

**Author’s response:** Thank you very much for your comment! We have made it clear in the revised manuscript. **(See the revised manuscript, Introduction section)**

Page 3, line 63: “In developing nations…”
- I suggest an alternative term to “developing”. E.g. resource-limited, low- and middle-income, Global South etc

**Author’s response:** Thank you very much for your critical comment! We have corrected it. **(See the revised manuscript, Introduction section)**

Page 3, lines 67-68: “The effectiveness of STI prevention and control heavily relies on the proactive health-seeking behavior exhibited by individuals dealing with these health challenges

- Although treatment-seeking is somewhat important in STI prevention and control, this statement arguably overstates its significance in STI prevention and control more broadly. Treatment-seeking is quite downstream within STI transmission. Suggest at minimum a reference to support this statement, if not re-phrasing to emphasise the treatment-seeking is just one factor.

**Author’s response:** Thank you very much for your professional suggestion! We have addressed it. **(See the revised manuscript, Introduction section)**

Page 4, lines 71-73: “As a result of the mild or absent symptoms experienced by the majority of men with STIs, they often choose not to seek treatment at public health facilities and may instead turn to self-medication.”
- If there are no symptoms, by definition the men will not seek treatment (including self-medication).

**Author’s response:** Thank you for your constructive comment! We have made it clear in the revised manuscript. **(See the revised manuscript, Introduction section**)

Page 4, lines 81-83: “Therefore, this study aimed to assess the pooled prevalence of STIs, related care-seeking behavior, and associated factors among sexually active men in East Africa.”
- I think there is a typo here – should this not read “this study aimed to assess the pooled prevalence of STI related care-seeking behavior, and associated factors among sexually active men in East Africa.”

**Author’s response:** Thank you for your professional insight. We have made corrections based on your comment. **(See the revised manuscript, Introduction section)**

Methods

- More information is required on DHS survey methodology, and particularly if there were any methodological differences between countries or survey years.

**Author’s response:** Dear reviewer thank you for your question. DHS survey methodology is similar across countries and survey years. As we have indicated in the study design and setting under our method section, DHS sample designs are usually two-stage probability samples drawn from an existing sample frame, generally the most recent census frame. Typically, DHS samples are stratified by geographic region and by urban/rural areas within each region. Within each stratum, the sample design specifies an allocation of households to be selected. Most DHS surveys use a fixed take of households per cluster of about 25-30 households, determining the number of clusters to be selected. In the first stage of selection, the primary sampling units (PSUs) are selected with probability proportional to size (PPS) within each stratum. The PSUs are typically census enumeration areas (EAS). The PSU forms the survey cluster. In the second stage, a complete household listing is conducted in each of the selected clusters. Following the listing of the households a fixed number of households is selected by equal probability systematic sampling in the selected cluster. Additional information on the DHS methodology is available at <https://dhsprogram.com/Methodology/index.cfm>

Data source and study population

- This paragraph could be more clear and the numbers don’t add up for me. I suggest stating explicitly here which eight countries are included in the present manuscript. Then state which countries have a history of DHS involvement, but which were not included (and the reasons for non-inclusion). Then state the number of countries with no history of DHS involvement.

**Author’s response:** Dear reviewer thank you for your comment. We have stated the countries, which are included in our study and the reasons for the exclusion of other East African countries.

Dependent variable

- More information needs to be provided on how a “STI” or “symptoms of an STI” were defined in the survey. For example, was it a set of defined symptoms?

**Author’s response:** Dear reviewer thank you for your comment. The DHS survey defined STI as the “Percentage of men aged 15-49 who had an STI in the past 12 months, had an abnormal genital discharge in the past 12 months, had a genital sore or ulcer in the past 12 months, had an STI or symptoms of an STI in the past 12 months”

Independent variables

- Justification for why these variables were chosen would be beneficial, potentially as supplementary material.

**Author’s response:** Dear reviewer thank you for your suggestion. We have chosen the potential predictors of the outcome variable based on the reviewed works of literature and their clinical importance.

Data processing and statistical analysis

- More information is needed on the exact model development process including the rationale for having multiple models

**Author’s response:** Dear reviewer thank you for your suggestion. As you know DHS data has a hierarchical nature, which means variables are measured at different levels like individual level and community level. We fit a null model without explanatory variables to determine the extent of cluster variation in healthcare-seeking behavior. We have also fitted a model with individual-level variables only, community-level variables only, and both individual and community-level variables to see which model will explain our data better.

Page 7, lines 136-138: “Bivariable multilevel binary logistic regression analysis was performed to identify variables eligible for the multivariable analysis at a p-value < 0.20.”

- Please present the results of these analyses, either in the results section or as supplementary material.

**Author’s response:** Dear reviewer thank you for your suggestion. We have included the bi-variable analysis results as a supplementary file.

Results

Page 8, line 159: “Among the 3,302 sexually active men who had STI in the past 12 months…”

- Adding up the total numbers for the surveys listed in figure 1 gives a total of 3101 participants, so it is unclear where the figure 3302 comes from – please clarify

**Author’s response:** Dear reviewer thank you for your suggestion. In the realm of descriptive statistics, we have utilized a weighted sample. This approach has assigned a weight to each observation in our dataset, accounting for its importance or representativeness. However, when generating a forest plot to estimate the pooled prevalence, we did not use a weighted sample. This was because the meta-analysis itself provides a weight for each country, which subsequently generates the pooled estimate.

- If possible, please provide information on number of people approached for surveys and declined etc to give a sense of how selection and other biases may have contributed. If this data is not available, please state this as a limitation.

- As above, need to define “men who had STI”

**Author’s response:** Dear reviewer thank you for your suggestion. However, the DHS data do not give any information regarding the number of individuals approached for interviewee and their response rate. We will acknowledge this in the limitation section

Page 8, line 162: “72.95% started sex before the age of 19”

- I suggest “had sex” rather than “started sex”, as the latter implies that this is ongoing

**Author’s response:** Dear reviewer thank you for your suggestion. We have used “had sex”

- Also, it would be helpful to have a definition of “sex” in the methods – does it refer solely to penile-vaginal penetrative intercourse?

**Author’s response:** Dear reviewer thank you for your question. DHS does not provide a definition of “sex”.

- Please provide a definition of “media exposure” in the methods

**Author’s response:** Dear reviewer thank you for your suggestion. We have provided an operational definition for the variable media exposure.

Table 1

- If there is no missing data, apart from the “comprehensive knowledge about HIV/AIDS” variable, I suggest including “n=3302 unless otherwise stated” in the title

**Author’s response:** Dear reviewer thank you for your suggestion. We have included the total sample size used in the current study which is n=3302 in the title.

- Please provide information in the methods on how the wealth index was derived

**Author’s response:** Dear reviewer thank you for your question. We have included how the wealth index is computed by the DHS in the method section.

- The variable “number of sex partners excluding spouse, in the last 12 months” is a slightly unusual variable as it assumes that sex with a spouse is equivalent to no sex in terms of risk. I don’t think the variable needs to be amended, but would suggest a justification for its use in the methods

**Author’s response:** Dear reviewer thank you for your question. The variable “number of sex partners excluding spouse, in the last 12 months” is not equivalent to no sex rather it's one indicator of risky sexual behavior, multiple sexual partners,

- “Covered by health” – I think this presumably should read “covered by health insurance”

**Author’s response:** Dear reviewer thank you for your question. We have corrected the editorial mistake.

Figure 1

- Please provide more information in the methods on how “pooled prevalence” was actually calculated

**Author’s response:** Dear reviewer thank you for your suggestion. We conducted a meta-analysis by combining prevalence estimates from different countries to obtain a pooled estimate. This statement has been included in the method section.

Table 2

- Please include p values

**Author’s response:** Dear reviewer thank you for your suggestion. We have included a p-value for each estimate.

Determinants of STI-related health care-seeking behavior among sexually active men in East Africa

- As stated above, more information needs to be provided on model development. In particular, what were the univariate associations of the independent variables with the dependent variable.

**Author’s response:** Dear reviewer thank you for your suggestion. We have included the univariate analysis result as a supplemental file.

Page 12, line 203: “…compared to men who reside in Zambia (Table 2).”

- Why was Zambia chosen as the reference category?

**Author’s response:** Dear reviewer thank you for your question. Zambia was chosen as a reference category because the country has the largest sample size compared to the others.

Discussion

Page 14, lines 214-216: “The societal norms and cultural practices in East Africa further reinforce the stigma associated with STIs, leading to a higher prevalence of untreated STIs among women in comparison to men.”

- Given that globally, STI prevalence is higher in women than men, this can unlikely be attributed to "the societal norms and cultural practices in East Africa" – please consider re-phrasing/removing

- If keeping this statement, please specify which societal norms and cultural practices are being referred to and provide a reference.

**Author’s response:** Dear reviewer thank you for your suggestion. We have explained the potential factors that contribute to the high prevalence of untreated STIs among women compared to men. We have highlighted potential societal norms and cultural practices like Stigma and Shame, Traditional Healers and Alternative Medicine, and Cultural Practices around Sexuality and Virginity. We have cited references as well. Please see the revised manuscript.

Page 14, lines 216-217: “As a result, it is crucial to develop effective interventions that tackle the social stigma associated with STIs in East Africa to encourage women to seek appropriate medical care.”

- Although true, I don’t this is really relevant to the current paper

**Author’s response:** Dear reviewer thank you for your suggestion. We have removed the statement

Page 14, lines 223-226: “The disparity with Chinese males could be attributed to a difference in the study population, as the study was conducted on men who had intercourse with men.”

- Men who have sex with men (MSM) is the more widely used term

- I think the men in the Chinese study being MSM is a really important distinction that significantly affects comparison as these are two very different cohorts – I would strongly recommend mentioning that these were MSM on first mention of the paper (on line 211), and compare and contrast the differences in these populations more thoroughly.

**Author’s response:** Dear reviewer thank you for your suggestion. We have mentioned MSM first and provided justification for the observed discrepancies regarding seeking treatment for STIs.

Page 15, lines 241-242: “Researchers with the CDC, who have some intriguing thoughts, support this.”

- I don’t think that references 28 and 29 are appropriate. I would recommend finding supporting evidence from peer-reviewed articles rather than blog posts.

**Author’s response:** Dear reviewer thank you for your suggestion. We have cited a research article

Page 15, lines 245-246: “Furthermore, married men have more financial means and are likelier to have health insurance than unmarried men (28,29).”

- Rather than relying on data from other papers, I would suggest analysing this within your dataset as a post-hoc analysis, as you have both marital and health insurance data.

**Author’s response:** Dear reviewer thank you for your suggestion. We have computed Tukey’s adjustment (Tukey’s Honestly Significant Difference (HSD) test) to compute p-values and confidence intervals for the pairwise differences. The comparisons are made over the variable marital status. This means that the means of wealth index and health insurance are compared across different categories of marital status. A positive value indicates that the mean of the health insurance and wealth index for the ever-married group is higher than that for the never-married group.

Page 16, lines 257-259: “Such a finding points to the need for the healthcare sector to enhance its counseling and awareness efforts during HIV/AIDS testing, thereby increasing the likelihood that women will seek care for STIs (31).”

- I am not entirely clear how the results relate to women seeking care for STIs?

**Author’s response:** Dear reviewer thank you for your question. It is an editorial mistake we have corrected it.

Strengths and limitations of the study

- An important limitation that is not mentioned is the reliance on secondary data and the effects this has on data interpretation.

- Additionally, please describe how inclusion of data from different time periods may have affected analysis. In particular, Rwanda’s DHS survey data was from 2019/2020 and Madagascar was from 2021, and so both may have been impacted by COVID-19.

**Author’s response:** Dear reviewer thank you for your suggestion. We have acknowledged the potential effect of differences in periods of the survey.

Conclusion

- As per comments on abstract conclusions

**Author’s response:** Dear reviewer thank you for your suggestion. We have revised our conclusion and include further explanations.

**Reviewer #2**

Reviewer #2: Title: Determinants of care-seeking behaviour for sexually transmitted infections among sexually active men in East Africa: a multilevel mixed effect analysis
Comments
Abstract
- How does the asymptomatic nature of STI relate to care-seeking behaviour among men? And based on this your introduction section do not clearly to show the gap

**Authors’ response**: We thank the reviewer for this valuable notice! We have made corrections in the revised manuscript. **(See the revised manuscript, introduction section of the Abstract)**

- Recent Demographic and Health 35 Surveys (DHSs) in East Africa: Which is recent?

**Authors’ response:** Thank you very much for your question. In the method section we have stated that we included surveys from 2011 to 2021.

- Is only the deviance value used to select the best model?

**Authors’ response:** Thank you very much for your question. We have used AIC and BIC in evaluating the goodness of fit of the statistical models. Nonetheless, when dealing with nested models, Deviance emerges as the most reliable test as it remains unaffected by the number of covariates and model complexity. It is important to note that the results of both AIC and BIC firmly indicate that the selected model is the best fit.

- In a result please include the number of successes, you included just the percentage.

**Authors’ response:** Thank you very much for your suggestion. The total number of successes is indicated in the forest plot (Figure 1).
- Present the result as a positive association and negative

**Authors’ response:** Thank you very much for your valuable comment! We have corrected it. **(See the revised manuscript, result section of the abstract)**

- Your abstract conclusion is just recommendations…..it is a good recommendation but you didn’t conclude the abstract, please include a concluding statement, main factors, and practical recommendations in sequence

**Authors’ response:** Thank you for your professional insight. We have made corrections based on your comment. **(See the revised manuscript, conclusion section of the abstract)**
Introduction
- Your introduction should have focused on care-seeking behaviour. This makes it look like a disorganized introduction. You may need to rearrange or add additional literature that adds more value to how health-seeking behavior could affect the overall effort.

**Author’s response:** Thank you very much for your professional suggestion! We have made amendments based on your suggestion. **(See the revised manuscript, Introduction section)**

- The introduction also lacks strong arguments that could have been based on the available statistics to show how East Africa and sub-Saharan regions are behind compared to others. It is only possible to speak about possible factors that may affect the region. As men are the most educated segment of the population, you need a very good argument about how the gap could happen.

**Authors’ response:** Thank you for your critical comments! We have completely accepted your concern, and we have revised it thoroughly. **(See the revised manuscript, Introduction section)**

Comments

Results

- You have very large result presentations; you may need to make a summary of results at the end

**Author’s response:** Dear reviewer thank you for your suggestion. Since the abstract section already presents a summary of the results, we believe that summarizing them again at the end would be redundant. However, if you would like us to do so, we are willing to comply.

- Please, be more explicit in describing the results, how figure 1 prepared is not clear

**Author’s response:** Dear reviewer thank you for your question. Figure 1 is a forest plot indicating the pooled prevalence of SIT-related care-seeking behavior. We conducted a meta-analysis by combining prevalence estimates from different countries to obtain a pooled estimate. This statement has been included in the method section.

Discussion

- Result comparison could make more sense if you can make a more focused comparison with similar setups or regions and reasonably compare how that is common is most developing countries. Something is missing in your initial paragraph –please include your reflection and your influence on the research which may caused some problems in the limitation section

**Author’s response:** Dear reviewer thank you for your suggestion. Because there are a limited studies done in Africa we couldn’t find enough studies for comparison.

Conclusion

- I already commented on how to make the conclusion more practical earlier, thus, apply here.

**Author’s response:** Dear reviewer thank you for your suggestion. We have revised the conclusion as per your suggestion
