# Supplementary material for: Determinants of care-seeking behavior for sexually transmitted infections among sexually active men in East Africa: A multilevel mixed effect analysis
Source: PLoS One. 2024 Sep 5;19(9):e0307755. doi: 10.1371/journal.pone.0307755 (PMC11376515; doi:10.1371/journal.pone.0307755)
Supplement: Attachment — Submitted filename: point by point response.docx [file pone.0307755.s003.docx]

**2.** My comment was: “Page 3, lines 53-54: “Globally, the prevalence of sexually transmitted infections (STIs) is rising despite the adoption of effective preventive measures”- The phrase "despite the adoption of effective preventive measures" is both broad and vague. It is unclear what preventive measures are being referred to. At minimum, a reference is required to support this statement.
- Author’s response: Thank you very much for your comment! We have made it clear in the revised manuscript. (See the revised manuscript, Introduction section)
- All the authors have changed “effective preventive measures” to “typically effective and financially feasible preventive measures”. I think this has made this statement more confusing. Please specifically state what preventive measures are being referred to e.g. “despite the adoption of preventive measures such as XXXX, XXXX and XXX”. The reason I feel so strongly about this is that many resource-limited settings rely on syndromic management for STIs, which is a relatively ineffective STI control measure, and also STI control has historically been underfunded (especially in comparison to HIV). At present, it is unclear what "effective" or "typically effective" measures the authors refer to.

**Author’s response:** Thank you very much for your feedback! We have taken your suggestion into consideration and have made the necessary revisions for greater clarity. In the revised manuscript, we now specify the preventive measures being referred to. The revised statement in the Introduction section now reads:

" The global prevalence of sexually transmitted infections (STIs) is rising despite the adoption of preventive measures such as comprehensive sexual education, condom distribution programs, regular screening and testing, partner notification and treatment, and public awareness campaigns."

We believe this revision addresses your concern by clearly listing specific preventive measures, making the statement more precise and informative.

**3.** I suggested that the authors should the paper by Ogale et al. (10.1371/journal.pgph.0001626) given its high relevance to the present manuscript. Although the authors included it as a reference, there has been no inclusion of the paper in terms of discussing the findings and comparing and contrasting the results. As mentioned previously, this should be a key focus of comparison in the discussion.

**Author’s response:** Thank you for your insightful critique. We acknowledge the significance of the study conducted by Ogale and colleagues. However, due to the distinct findings between their study and ours, we have made a deliberate choice not to incorporate their specific findings into our discussion. This decision stems from the distinct focus and objectives of our study, which differ from those of Ogale et al. As such, incorporating their results would potentially detract from our study's intended scope and disrupt the coherence of our overall research concept.

**4.** Page 4, lines 74-76: “The effectiveness of STI prevention and control significantly relies on individuals' proactive health-seeking behavior when facing these challenges, recognizing that treatment seeking is one factor among several contributing to this effectiveness.”
- I appreciate that the authors amended the above sentence to note that treatment-seeking behaviour is just one factor in overall STI prevention and control. However, it is worded quite awkwardly. Suggest re-phrasing to something like “Treatment seeking behaviour is an important factor in contributing to the overall effectiveness of STI prevention and control”

**Author’s response:** Thank you for your valuable comment! We have rephased it in the revised manuscript. **(See the revised manuscript, page 3, line 75-77, Introduction section)**

**17.** Page 2, lines 34-35: “The success of preventing and controlling STIs largely depends on how actively individuals seek healthcare when dealing with these issues.”
- I disagree with the phrasing of this sentence. “Largely depends” suggests an outsized effect. Suggest re-phrasing to “The treatment seeking behaviour of individuals in relation to STIs is an important factor in STI prevention and control”.

**Author’s response:** Thank you for your constructive comment and professional suggestion. We have made changes as per your suggestion. **(See the revised manuscript, page 2, line 34-35, Background section of Abstract)**

**18.** Page 2, lines 51-52: “Age, marital status, number of sexual partners, ever 51 been tested for HIV, and media exposure, were identified as factors associated with healthcare-seeking behavior for STIs.”
- I appreciate this was an edit in response to reviewer 2’s suggestion. However, I don’t think you should just repeat the results in such a manner. Consider highlighting the key associations and messages without just repetition of results.

**Author’s response:** Dear reviewer thank you for your professional insight! We have made amendments based on your as well as second reviewer’s comments**. (See the revised manuscript, page 2-3, line 50-53, Conclusion section of Abstract)**

**Reviewer #2:** The conclusion is not improved, it is probably worsened. In my last comment, I pleaded with you to include a concluding statement(1st position), main factors, and practical recommendations in sequence. After all, this is the main section that should give clues for what, overall view, what was identified, and what was missing.
The introduction section is well improved but still not supported by numerical evidence or magnitude of the problem in the region.

**Author’s response:** Thank you for your valuable insights and suggestions. We have taken into account your suggestions, along with those from the first reviewer, and have made revisions accordingly. **(See the revised manuscript, page 2-3, line 50-53, Conclusion section of Abstract)**

Methods

Data source and study population

5. My original comment was: “This paragraph could be more clear and the numbers don’t add up for me. I suggest stating explicitly here which eight countries are included in the present manuscript. Then state which countries have a history of DHS involvement, but which were not included (and the reasons for non-inclusion). Then state the number of countries with no history of DHS involvement.”

- Unfortunately no changes have been made in response to this suggestion. Firstly, please state in this paragraph what countries are included.

- Secondly, as stated above, the reason I requested for more detail to be provided was that the numbers do not make sense. If 14 countries have a documented history of involvement in the DHS, then to have 8 countries included there must be 6 that have other reasons for non-inclusion. However, 7 such countries are reported (Sudan, Eritrea, Uganda, Mozambique, Malawi, Tanzania, Kenya).

**Author’s response:** dear reviewer thank you very much for your detailed comment. We have made corrections based on your suggestion. **(See the revised manuscript, lines 110-116, page 5, methods section)**

Dependent variable

6. My original comment was: “More information needs to be provided on how a “STI” or “symptoms of an STI” were defined in the survey. For example, was it a set of defined symptoms?

- The author’s response stated “The DHS survey defined STI as the “Percentage of men aged 15-49 who had an STI in the past 12 months, had an abnormal genital discharge in the past 12 months, had a genital sore or ulcer in the past 12 months, had an STI or symptoms of an STI in the past 12 months”

- Please include this definition in the methods

**Author’s response:** Dear reviewer thank you for your suggestion. We have included the definition in the method section. **(See the revised manuscript ,Page 6, line 120-122 method section).**

Independent variables

7. My original comment was: Justification for why these variables were chosen would be beneficial, potentially as supplementary material.

- The authors state that “We have chosen the potential predictors of the outcome variable based on the reviewed works of literature and their clinical importance.”

- This justification should be included in the manuscript, alongside references supporting their inclusion

**Author’s response:** Dear reviewer thank you for your suggestion. We have included the statement with respective references in the method section. **(See the revised manuscript, Page 6, line 133-134 method section).**

Data processing and statistical analysis

8. My original comment was: “More information is needed on the exact model development process including the rationale for having multiple models”

- In their comment the authors provided some justification by stating “We have also fitted a model with individual-level variables only, community-level variables only, and both individual and community-level variables to see which model will explain our data better.”

- However, such justification must be included in the manuscript

**Author’s response:** Dear reviewer thank you for your suggestion. We have included the statement with respective references in the method section. **(See the revised manuscript, Page 7, line 167-171 method section).**

Results

9. My original comment was: “Page 8, line 159: “Among the 3,302 sexually active men who had STI in the past 12 months…” - Adding up the total numbers for the surveys listed in figure 1 gives a total of 3101 participants, so it is unclear where the figure 3302 comes from – please clarify

- Thank you to the authors for providing clarity as to why the numbers differ. Please make this clear in the methods so that future readers do not also find it confusing.

**Author’s response:** Dear reviewer thank you for your suggestion. We have included a clarification for discrepancies in the figure and table. **(See the revised manuscript, Page 7, line 156-160 method section).**

10. My original comment was: “If possible, please provide information on number of people approached for surveys and declined etc to give a sense of how selection and other biases may have contributed. If this data is not available, please state this as a limitation.”

- The authors response was: “The DHS data do not give any information regarding the number of individuals approached for interviewee and their response rate. We will acknowledge this in the limitation section”

- I cannot see the inclusion of this in the limitations section. Please ensure it is included.

**Author’s response:** Dear reviewer thank you for your suggestion. We have acknowledged the limitation in the strength and limitation section. **(See the revised manuscript ,Page 17, line 311-313 method section).**

Figure 1

11. My original comment: “Please provide more information in the methods on how “pooled prevalence” was actually calculated

- Author’s response: Dear reviewer thank you for your suggestion. We conducted a meta-analysis by combining prevalence estimates from different countries to obtain a pooled estimate. This statement has been included in the method section.

- I am unable to find any such statement included in the methods section

**Author’s response:** Dear reviewer thank you for your suggestion. We have included the statement in the method section of data processing and statistical analysis section. **(See the revised manuscript, Page 7, line 161-163 method section).**

Table 2

12. Thank you to the authors for including p values in table 2 following my suggestion.

- However, I would recommend a pooled p-value for each variable (e.g. a p-value for age, rather than one for each age group).

- I apologise for not making this more clear in the original comments.

**Author’s response:** Dear reviewer thank you for your suggestion. We have included the p-value in the revised regression table.

- In their responses, the authors discuss a supplemental file. However, this was not uploaded with the current paper. Nor is their any reference to it in the manuscript

**Author’s response:** Dear reviewer thank you. we have included the supplementary file with its respective reference in the main document. **(See the revised manuscript, Page 8, line 166-167 method section).**
